# Area of exposure and treatment challenges of malaria in Eritrean migrants: a GeoSentinel analysis

**DOI:** 10.1186/s12936-018-2586-9

**Published:** 2018-11-29

**Authors:** Patricia Schlagenhauf, Martin P. Grobusch, Davidson H. Hamer, Hilmir Asgeirsson, Mogens Jensenius, Gilles Eperon, Camilla Rothe, Egon Isenring, Jan Fehr, Eli Schwartz, Emmanuel Bottieau, Elizabeth D. Barnett, Anne McCarthy, Paul Kelly, Carsten Schade Larsen, Perry van Genderen, William Stauffer, Michael Libman, Philippe Gautret

**Affiliations:** 1 WHO Collaborating Centre for Travel Medicine, Travel Clinic and Department of Public Health, Epidemiology, Biostatistics and Prevention Institute, University of Zürich, 8001 Zurich, Switzerland; 2 Centre for Tropical Medicine and Travel Medicine, Department of Infectious Diseases, Academic Medical Centre, University of Amsterdam, Amsterdam, The Netherlands; 3 Department of Global Health, Boston University School of Public Health and Section of Infectious Diseases, Department of Medicine, Boston University School of Medicine, Boston, MA USA; 4 Department of Infectious Diseases, Karolinska University Hospital, Stockholm, Sweden; 5 Unit of Infectious Diseases, Department of Medicine Huddinge, Karolinska Institutet, Stockholm, Sweden; 6 Department of Infectious Diseases, Oslo University Hospital, Oslo, Norway; 7 Division of Tropical and Humanitarian Medicine, Department of Community Medicine, Primary and Emergency Care, Geneva University Hospitals (HUG), Geneva, Switzerland; 8 Division of Infectious Diseases and Tropical Medicine, LMU University Hospital Munich, Munich, Germany; 9 University Hospital, Department of Infectious Diseases, University of Zürich, Zurich, Switzerland; 10 The Center of Geographical Medicine-Dept. of Internal Medicine “C”-Sheba Medical Center Tel HaShomer, and Sackler Faculty of Medicine, Tel Aviv University, Tel Aviv, Israel; 11 Department of Clinical Sciences, Institute of Tropical Medicine, Antwerp, Belgium; 12 Maxwell Finland Laboratory for Infectious Diseases, Boston Medical Center, Boston, MA USA; 13 Ottawa Hospital and Department of Medicine University of Ottawa, Ottawa, Canada; 14 Bronx Lebanon Hospital, New York, USA; 15 Department of Infectious Diseases, Aarhus University Hospital, Aarhus, Denmark; 16 Institute for Tropical Diseases, Harbour Hospital Rotterdam, Rotterdam, The Netherlands; 17 Infectious Diseases and International Medicine, University of Minnesota, Minneapolis, USA; 18 J.D. MacLean Centre for Tropical Diseases, McGill University, Montreal, Canada; 19 University Hospital Institute for Infectious and Tropical Diseases, Aix-Marseille University, Marseille, France

## Abstract

**Background:**

Recent reports highlight malaria as a frequent diagnosis in migrants who originate from Eritrea. A descriptive analysis of GeoSentinel cases of malaria in Eritrean migrants was done together with a literature review to elucidate key attributes of malaria in this group with a focus on possible areas of acquisition of malaria and treatment challenges.

**Results:**

A total of 146 cases were identified from the GeoSentinel database from 1999 through September 2017, with a marked increase in 2014 and 2015. All patients originated from Eritrea and the main reporting GeoSentinel sites were in Norway, Switzerland, Sweden, Israel and Germany. The majority of patients (young adult males) were diagnosed with malaria following arrival in the host country. All patients had a possible exposure in Eritrea, but may have been exposed in documented transit countries including Ethiopia, Sudan and possibly Libya in detention centres. Most infections were due to *Plasmodium vivax* (84.2%), followed by *Plasmodium falciparum* (8.2%). Two patients were pregnant, and both had *P. vivax* malaria. Some 31% of the migrants reported having had malaria while in transit. The median time to onset of malaria symptoms post arrival in the host country was 39 days. Some 66% of patients were hospitalized and nine patients had severe malaria (according to WHO criteria), including five due to *P. vivax*.

**Conclusions:**

The 146 cases of mainly late onset, sometimes severe, *P. vivax* malaria in Eritrean migrants described in this multi-site, global analysis reflect the findings of single-centre analyses identified in the literature search. Host countries receiving asylum-seekers from Eritrea need to be prepared for large surges in vivax and, to a lesser extent, falciparum malaria, and need to be aware and prepared for glucose-6-phosphate dehydrogenase deficiency testing and primaquine treatment, which is difficult to procure and mainly unlicensed in Europe. There is an urgent need to explore the molecular epidemiology of *P. vivax* in Eritrean asylum-seekers, to investigate the area of acquisition of *P. vivax* along common transit routes and to determine whether there has been re-introduction of malaria in areas, such as Libya, where malaria is considered eliminated, but where capable vectors and Plasmodium co-circulate.

**Electronic supplementary material:**

The online version of this article (10.1186/s12936-018-2586-9) contains supplementary material, which is available to authorized users.

## Background

Conflict-related migration challenges health systems in host countries and population movements have an impact on infectious disease epidemiology. Migrants present to local medical facilities in host countries with unfamiliar infectious diseases that may require unlicensed treatments. These circumstances have implications for screening, diagnostics, medication procurement and adherence to treatment. Several reports have highlighted that malaria is a frequent diagnosis in migrants, who originate in Eritrea [1–3] and who transit countries in sub-Saharan and northern Africa. National malaria surveillance statistics show a marked increase in imported malaria in asylum seekers [3, 4]. Migrants from Eritrea encompass both refugees and asylum-seekers. An *asylum*-*seeker* is an individual who has sought international protection and whose claim for refugee status has not yet been determined [5]. Countries are responsible for determining whether an asylum-seeker is a refugee or not. Eritrea is considered to be one of the world’s “fastest emptying nations” mainly due to forced army conscription that may last a lifetime. Eritrea ranks 5th in the top ten origins of persons applying for asylum in the European Union [5].

The United Nations High Commissioner for Refugees (UNHCR) reported 474,296 Eritreans globally to be refugees and asylum-seekers [5]. This constitutes approximately 12% of Eritrea’s official 3.6 million population estimate as of 2015. Using the dual approach of a GeoSentinel global database descriptive analysis and an in-depth literature review, this analysis describes malaria in Eritreans presenting at GeoSentinel sites and evaluates the complexity of presentation and treatment in this distinct group of migrants.

## Methods

GeoSentinel (http://www.geosentinel.org) sites are clinics specializing in travel, migration and/or tropical medicine, that contribute clinician-based data on ill migrants and travellers to a global database. To be eligible for inclusion in the database, the patient must have crossed an international border in the 10 years prior to presentation and the diagnosis must be considered “travel-related” by the reporting GeoSentinel clinician [6]. GeoSentinel’s data collection protocol has been reviewed by the institutional review board officer at CDC’s National Center for Emerging and Zoonotic Infectious Diseases and is classified as public health surveillance and not human subject research. When indicated by national regulations at individual GeoSentinel sites, additional ethical clearance has been obtained.

Only symptomatic Eritrean migrants with *confirmed* malaria diagnoses were included in this analysis. Clinical data captured included the presenting symptoms, malaria species identification, severity of infection and in-patient/out-patient status. ‘*Severe malaria*’ was defined according to World Health Organization (WHO) criteria [7]. Demographic data were also collated: age, sex, country of birth, and country of residence/asylum, travel history (arrival date and transit routes when available). GeoSentinel sites use the best reference diagnostic tests available and screen for glucose-6-phosphate dehydrogenase (G6PD) deficiency; the malaria cases in this study were based on presence of malaria symptoms and confirmation by microscopy or PCR or RDT.

In addition to the descriptive analysis of the GeoSentinel database, a literature review was done. The databases PubMed, Embase and Scopus were searched using the word combinations “malaria”, “migrant”, “Eritrea”. Articles in English, German, Italian and French were included. Relevant reports from public health agencies, ministries and malaria surveillance statistics were also screened. The time period for the database searches was January 1st 2010 to July 31st 2018. GeoSentinel Sites that contributed cases to this analysis, were queried regarding the availability of primaquine and G6PDH deficiency testing at their sites.

## Results

**Fig. 1:**
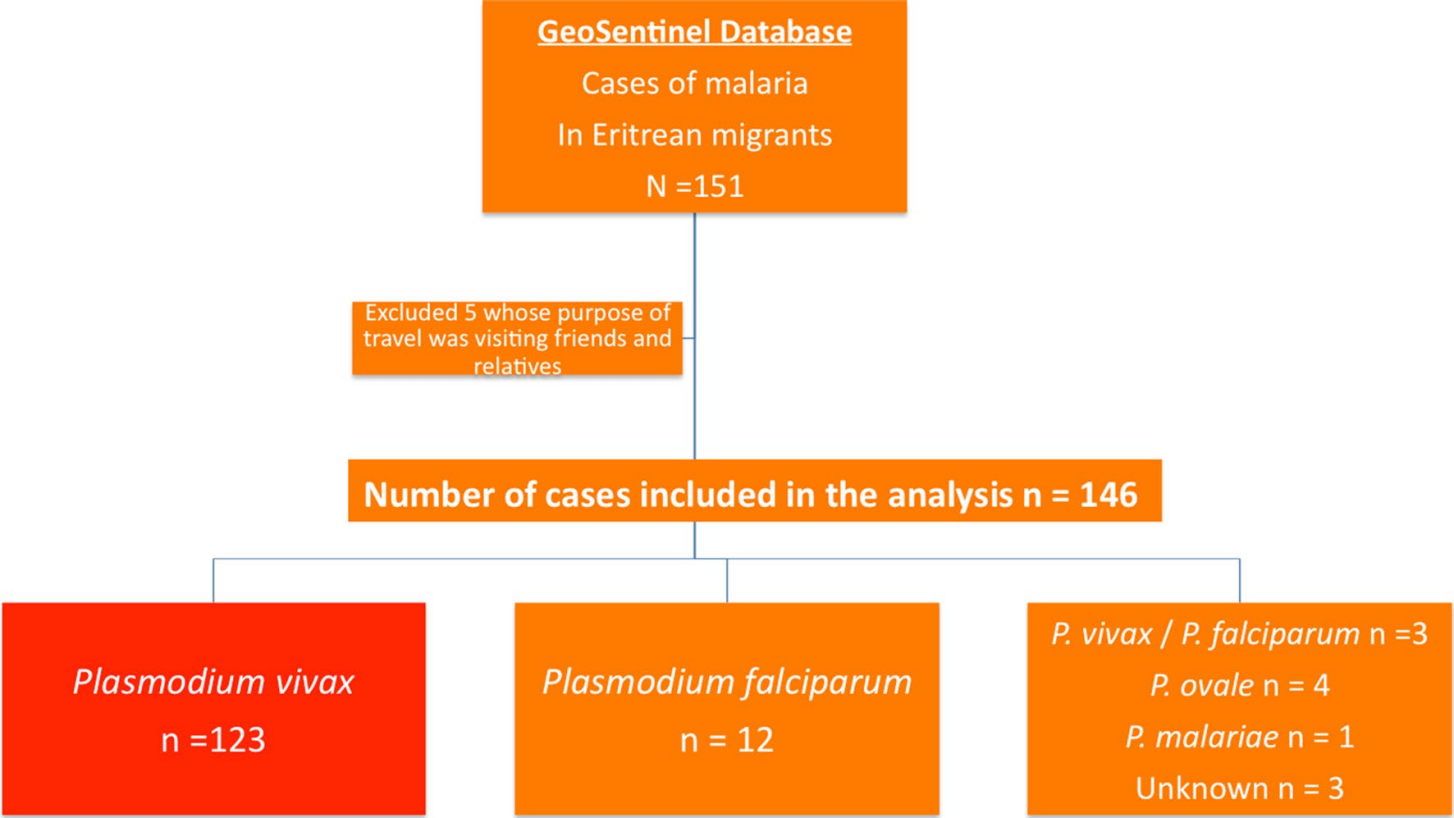
Flow chart of the GeoSentinel “Malaria in Eritrean Migrants” analysis

There were 146 cases identified from the GeoSentinel database from 1999 through September 15, 2017 (Fig. 1), with a marked increase in 2014 and 2015. There was a 66% hospitalization rate and 9 cases of severe malaria. No death was recorded. All patients originated from Eritrea and were diagnosed with malaria following their initial migration trip with the main reporting sites in Norway, Switzerland, Sweden, Israel and Germany (Table 1) in descending order of contribution. The majority of the patients were young adult males. The migration route was not documented for all patients (Table 2), but most of them were possibly also exposed in other transit countries, including notably Sudan 76% (41/54) and Ethiopia 57% (31/54). Additionally, 34 patients 63% (34/54) declared having transited Libya during their migration trip, 7 transit routes led through Egypt, one through Syria, one through Turkey and one through Uganda. Most infections were due to *Plasmodium vivax* (84.2%), followed by *Plasmodium falciparum* (8.2%), *Plasmodium ovale* (2.7%), *Plasmodium malariae* (0.7%), and unspecified species or mixed infection (Table 3). Most patients had the onset of malaria symptoms after arriving in the host country (median 39 days) and consulted a median of 3 days following symptom onset. About a third (31%) of the migrants reported having had malaria while in transit. All patients were symptomatic and the most common presenting symptoms were fever (98.0%), gastrointestinal (9.6%), fatigue (8.2%), headaches (7.5%) and respiratory symptoms (6.9%). Two patients (aged 20 and 21 years, respectively) with *P. vivax* malaria were pregnant. Nine patients were reported to have severe malaria (Table 3), including: *P. vivax* infection with severe anaemia (n = 3), *P. vivax* infection with renal failure (n = 1), *P. vivax* infection with cardiovascular failure (n = 1), *P. falciparum* infection with severe anaemia (n = 1), *P. falciparum* infection with hyperparasitaemia and respiratory failure, unknown malaria species infection with neurological symptoms (n = 1) and unknown species infection with cardiovascular failure (n = 1). The survey of GeoSentinel sites showed that primaquine is unregistered and difficult to procure in Europe but available in the USA and Canada and that the speed of G6PDH deficiency testing is variable (Additional file 1). The literature review, identified 10 papers that refer to malaria in Eritrean migrants.

**Table 1:** Demographics

Country of origin	Eritrea malaria cases n = 146 (100.0%)
Country of immigration (Host country)	
Norway	35 (24.0%)
Switzerland	31 (21.2%)
Sweden	25 (17.1%)
Israel	19 (13.0%)
Germany	17 (11.6%)
Denmark	7 (4.8%)
Belgium	5 (3.4%)
UK	4 (2.7%)
USA	2 (1.4%)
Canada	1 (0.7%)
Gender	
Male	114 (78.1%)
Female	32 (21.9%)
M/F ratio	3.6
Age	
Mean (SD)	22.9 years (7.5 years)
Median (range)	22 years (4–55 years)
< 15 years	5 (3.4%)

**Table 2:** Transit and possible exposure countries

Possible country of exposure besides Eritrea (Transit countries)	
Sudan	43 (29.5%)
Libya	34 (23.3%)
Ethiopia	31 (21.2)
Egypt	7 (5.0%)
Syria	1 (0.7%)
Turkey	1 (0.7%)
Uganda	1 (0.7%)
Not documented	92 (63.0%)

**Table 3:** Clinical features of the malaria cases [all diagnoses were confirmed except in 4 patients (two *P. vivax* mono-infections were probable diagnoses, and in two mixed infections, *P. falciparum* infection was probable while *P. vivax* infection was confirmed)] in Eritrean migrants

Time of onset in relation to time of arrival in the host country^a^	
Onset of symptoms before arrival	31 (31.0%)
Onset of symptoms after arrival	69 (69.0%)
Time between arrival date and onset of symptoms in patients with onset after arrival (N = 69)	94.9 days (148.9)
Mean (standard deviation)	39 days (0–721)
Median (range)	
Time between onset of symptoms and visit date to GeoSentinel clinic (N = 69)	9.9 days (19.1)
Mean (standard deviation)	3 days (0–92)
Median (range)	
Location of care	
Inpatient	96 (65.8%)
Outpatient	50 (34.2%)
Malaria classification	
Non-severe	137 (93.9%)
Severe and complicated^b^	9 (6.1%)
Main symptoms	
Fever/sweats/chills	143 (98.0%)
Gastrointestinal	14 (9.6%)
Fatigue	12 (8.2%)
Headaches	11 (7.5%)
Respiratory	10 (6.9%)
Neurologic	5 (3.4%)
Genito-urinary/renal	3 (2.1%)
Cardiac	2 (1.4%)

^a^Information available for 100 patients out of 146

^b^*P. vivax* infection with severe anaemia (n = 3), *P. vivax* infection with renal failure (n = 1), *P. vivax* infection with cardiovascular failure (n = 1), *P. falciparum* infection with severe anaemia (n = 1), *P. falciparum* infection with hyperparasitaemia and respiratory failure (n = 1), unknown malaria species infection with neurological symptoms (n = 1) and unknown malaria species infection with cardiovascular failure (n = 1)

## Discussion

This descriptive analysis of malaria cases ex Eritrea notified to GeoSentinel shows that the majority of cases were *P. vivax* (84%) and the median time to presentation after arrival in the host country was 39 days (for those for whom data were available). These results mirror the findings of single centre studies in Europe [1–4, 8, 10–12, 32]. and Israel [2] and reflect a shift in imported malaria species dominance in Europe due to the changing demographics of migrants and asylum seekers. Prior to 2013, *P. falciparum* was the predominant malaria species imported to many European countries and was associated primarily with immigration from sub-Saharan Africa, mainly West Africa and, to a lesser extent, with returning travellers who had visited sub-Saharan Africa, but who failed to use chemoprophylaxis [9]. Since 2013, Eritrean asylum seekers have changed the profile of imported malaria cases in Europe with a shift towards *P. vivax*. In Germany, prior to 2013, 80% of all imported malaria cases, numbering 500–600 total cases annually were falciparum malaria cases, and vivax malaria was a minor contributor (approximately 7%) [3]. One study from a large Hamburg University hospital saw a relative increase in *P. vivax* malaria from only 2% of all cases in 2013, to 26% in 2014, and 34% in 2015. Approximately 95% of these vivax cases were in Eritreans [3]. In the Netherlands, during the period 2008–2015, *P. vivax* infections originated mainly from the Horn of Africa (214/372; 57.5%) and were largely attributable to asylum seekers from Eritrea [1]. In Milan, northern Italy, the proportion of *P. vivax* cases treated at a referral university hospital rose from 14.5% *P. vivax* in 2013 to 33.3% *P. vivax* in 2014 due mainly to malaria cases in Eritrean migrants [10]. In Sweden, a significant number of *P. vivax* cases in asylum-seekers from Eritrea led to the largest surge in imported malaria cases since computerized recording of malaria cases began in 1986 [4]. Because Sweden has accurate denominator data on asylum-seekers, the incidence rate of *P. vivax* in Eritreans was estimated at 19.5 per 1000 asylum seekers. In those aged less than 18 years, the incidence rate was considerably higher and estimated at 38.2 per 1000 [4].

### Time to presentation

**Fig. 2:**
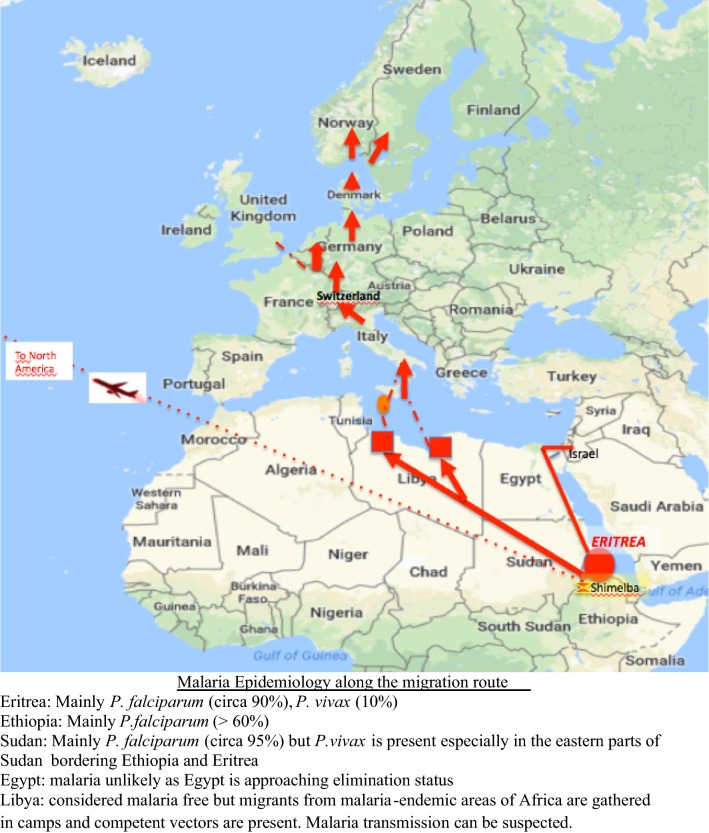
Map of migration routes from Eritrea overland to Europe and via airlift to the US and Canada and malaria epidemiology on the migration paths

The GeoSentinel analysis showed that about 69% of the migrants with malaria presented after arrival and the median time between arrival in the host country and presentation was 39 days (Table 2). Many of the Eritrean asylum-seekers had a long, arduous route to Europe that may have lasted several months (Fig. 2) and 31% reported malaria symptoms while in transit. These may have been *P. falciparum* cases or early onset *P. vivax*. Some strains of *P. vivax* do not produce primary attacks soon after infection and the first clinical symptoms may occur several months after the infective bite. Although there are few data on vivax time to relapse from Eritrea. *P. vivax* strains from the Horn of Africa have been recognized to have a long relapse interval [13, 14]. Compared to *P. falciparum*, regional populations of *P. vivax* exhibit high genomic diversity [15, 16]. The molecular epidemiology of *P. vivax* presenting in Eritrean migrants needs further investigation. Another result of late presentation and/or delayed diagnosis is an increased risk of severe malaria as evidenced here in this analysis with a 66% hospitalization rate and 9 cases of severe malaria. The difficulty of achieving an early, accurate diagnosis of vivax malaria, due to low sensitivity of both blood smear and malaria rapid diagnostic tests (RDTs) remains an issue in clinical practice in Europe and delayed diagnosis may have contributed to the high proportion of complicated cases, including the severe anaemia reported here.

### Where was malaria acquired—in Eritrea or in transit?

There are few data available on the epidemiology of malaria in Eritrea. Eritrea is located at the most northern limit of malaria transmission in East Africa. A parasitological, cross-sectional survey published in 2005 [17] documented screening and testing of 13,000 individuals in 176 villages in Eritrea. Malaria prevalence was low and focal and distributed across all age groups (suggesting low natural immunity) and *P. falciparum* was the dominant species (90%). One limitation of the aforementioned study was the low sensitivity and specificity of the OptiMal RDT used as the single screening test. Did the study miss the local *P. vivax* while preferentially documenting *P. falciparum*? An independent assessment in 2008 of OptiMal for *P. vivax* malaria found a sensitivity close to 95% for 200 parasites per microlitre [18] but depending on technique the sensitivity was as low as 75% and other studies report a much lower sensitivity, especially at low grade parasitaemia [19, 20]. Most patients with malaria seen in this study and in the studies identified during the literature review had *P. vivax*. This does not reflect the local epidemiology in Eritrea as described by Sintasath et al. [17] or that in the latest World Health Organization World Malaria Report that shows an approximate species prevalence of *P. falciparum* of 89% in Eritrea [21]. This suggests that the acquisition of malaria infection occurred later, most probably during the migration route, and that falciparum malaria tended to present, and presumably be treated, en route, prior to arrival in the host country. An Israeli report of *P. vivax* in Eritreans suggested that the most likely place of malaria exposure was in refugee camps in eastern Sudan [2]. In this analysis, at least 31 of the migrants/asylum seekers transited Ethiopia and this region may have been the source of some of the infections [22]. In seven cases, Egypt was included in the transit route and while this would be an unlikely area of acquisition (due to the current malaria elimination status) cross-border movements render the area vulnerable to re-introduction [23].

From 2000 through 2016 a total of approximately 17,050 Eritrean refugees and immigrants were admitted into the U.S, primarily resettled from camps (Shimelba and Mianey) in Ethiopia (Fig. 2). Although Eritrean by ethnicity, only 41% were born in Eritrea, with approximately 53% being born in Ethiopia, and Sudan accounting for the birth country for the majority of the remainder [24]. These refugees, unless they had a contraindication, received pre-departure anti-malarials prior to leaving Africa for presumptive treatment; since 2007, the pre-departure treatment is artemether/lumefantrine. Only two US malaria cases (both *P. vivax*) were identified in the GeoSentinel analysis despite large numbers of Eritreans resettled in the US suggesting that Eritreans with malaria are either not presenting at US GeoSentinel sites, or, more likely, are benefitting from pre-departure evaluations and mass drug administration with anti-malarials before direct resettlement to the USA via regularized migration pathways (Additional file 1).

This situation suggests that *P. vivax* occurred later in the transit route than the pre-US departure camps in Ethiopia because artemether/lumefantrine would not prevent the relapse vivax infections that were seen in the majority of the cases in Europe. These data highlight the importance of understanding the migration journey for all resettled migrants and the importance of efforts to provide pre-departure treatment at least for falciparum malaria. Furthermore, this supports the recommendation made at the United Nations General Assembly by Secretary General Gutierrez that efforts must be made to channel refugee movements toward regularized structured migration and away from chaotic irregular flows. This includes the provision of comprehensive health services for assessment, screening, treatment and prevention as early in the displacement process as possible.

Libya was also part of the transit route for 34 of the cases described here. There is some speculation regarding malaria transmission in Libya and hypothesized possible acquisition of *P. falciparum* malaria in Libyan detainment camps [25, 26]. In the Libyan camps, migrants are in close contact with other refugees including migrants from West Africa and it is plausible that malaria may be transmitted in these centres. This study identified cases of *P. falciparum* (8.2%) and *P. ovale* (2.7%) in the Eritrean migrants. *Anopheline* mosquitoes are present in Libya including *Anopheles multicolor*, *Anopheles sergentii* and *Anopheles labranchiae* [27]. These vectors are potentially capable of transmitting *P. vivax, P. falciparum* and *P. ovale*. Another concern further on in the transit route is that there is a potential for the reintroduction of malaria in receptive European areas. Multiple reports in 2017 have documented locally acquired malaria, both *P. vivax* and *P. falciparum*, in Greece, Cyprus, France and Italy and these have been summarized in an European Centre for Disease Control (ECDC) rapid risk assessment report [28]. Even further north in temperate Europe, the current situation of “Anophelism without malaria” may be threatened by large numbers of imported *P. vivax* malaria in the presence of competent vectors, such as *Anopheles atroparvus*, *Anopheles messeae* and *Anopheles sacharovi*. The capacity for dormancy has allowed *P. vivax* to be transmitted in Europe and North America and other temperate zones where the climate is normally prohibitive to transmission of other malaria species. Large outbreaks of vivax malaria have been reported in temperate zones when vivax infected individuals return to temperate areas after exposure in *P. vivax* endemic regions [29].

### G6PD deficiency testing, primaquine treatment and implications for clinical practice

Primaquine treatment, 15(− 30) mg base per day for 14 days, is indicated to prevent relapses by hypnozoite elimination. Primaquine is contraindicated in G6PD severely deficient individuals because of the risk of a potentially fatal haemolysis [30]. It is also controversial in imported malaria due to all the problems related to a considerable number of individuals being mildly-to-severely G6PD deficient; the adherence to the long-duration regimen as well as limited ad-hoc availability of the drug in many European countries (Additional file 2). The same restrictions will apply to the alternative 8-aminoquinoline, tafenoquine, when it becomes available [31]. It is considered standard of care to test for G6PD deficiency in all persons prior to use of primaquine. In case of severe G6PD deficiency, less than 10% residual enzymatic activity, primaquine should be avoided and the risk–benefit balance carefully assessed. In case of moderate deficiency, WHO proposes an 8-week schedule for treatment with primaquine: 0.75 mg/base/kg body weight once weekly for 8 weeks [32]. A practical problem is that the current reference method for G6PD testing is UV spectrophotometry. This method requires considerable laboratory infrastructure [33]. Inexpensive, highly reliable ‘field’, or point-of-care, tests are in development, but have not yet reached the level of development needed to enter the market [33]. Physicians dealing with imported cases of vivax malaria must strive to secure access to primaquine and to pre-empt relapses in their patients after having firmly established their G6PD status. Primaquine is widely available in Canada and in the USA; it is FDA-approved for the radical cure (prevention of relapse) of vivax malaria. In Switzerland and many EU countries, primaquine is only available through hospital pharmacies or through an international pharmacy with delays in procurement [34]. The availability of primaquine in is summarized in the Additional file 2.

### Malaria detection in the hard-to-reach migrant population, and possible policy implications

Migrant populations are considered “hard-to-reach” for infectious disease care [34, 35] in terms of use and access to regular health services and this applies to Eritreans who are displaced within prosperous nations in Europe. Early diagnosis and correct management of malaria in Eritrean migrants will prevent severe disease. The procurement logistics and long therapy duration with primaquine, the need for pre-treatment G6PD screening, in an Eritrean population with an allele prevalence of 3–7% [36] dictate that a specific policy is needed to address malaria in Eritrean migrants.

### Strengths and limitations

This is a large descriptive analysis of malaria cases in Eritrean asylum-seekers. A major strength of the GeoSentinel database is the global linkage of clinician verified malaria clinical data with accurate demographics and detailed travel history including transit routes. The improved GeoSentinel data collection form for migrants captures data elements on the status of the migrant (refugee, asylum-seeker) and screening results. GeoSentinel analyses constitute a valuable approach to documenting epidemiological patterns in mobile populations such as migrants. This GeoSentinel study has the limitation that denominator data are unavailable and that only a subset of all malaria cases in Eritrean migrants were captured. Because GeoSentinel sites in Europe are often important reference hospitals, this may have led to a bias selecting the more severe malaria cases and cases treated elsewhere are missed. Only two US cases (both *P. vivax*) were included in this analysis despite large numbers of Eritreans resettled in the US with 4685 individuals registered between October 1, 2016 and April 30, 2017, according to the Migration Policy Institute [24].

## Conclusions

This is the first multi-centre, global study of malaria in Eritrean migrants. It is the first study to provide clinical detail on malaria cases, demographics and itinerary data on Eritreans presenting at several clinics throughout Europe. It highlights the need for current or future host countries receiving asylum-seekers from Eritrea to be prepared for large surges in vivax malaria. The treatment of *P. vivax* will require complex pre-screening for G6PD deficiency and primaquine treatment. This study found that primaquine was difficult to procure throughout Europe and only licensed in North America.

Most importantly, this study highlighted the need explore the molecular epidemiology of *P. vivax* in Eritrean asylum-seekers, to investigate the area of acquisition of malaria along common transit routes and to determine whether there has been re-introduction of malaria in areas, such as Libya, where malaria is considered eliminated but where capable vectors and plasmodia co-circulate.

## Additional files

**Additional file 1.** Guidelines for pre-departure presumptive malaria treatment for refugees resettling to the USA from sub-Saharan Africa.

**Additional file 2.** Availability of primaquine and G6PDH deficiency testing at GeoSentinel sites.
