# Supplementary material for: Area of exposure and treatment challenges of malaria in Eritrean migrants: a GeoSentinel analysis
Source: Malar J. 2018 Nov 29;17:443. doi: 10.1186/s12936-018-2586-9 (PMC6267801; doi:10.1186/s12936-018-2586-9)
Supplement: Supplementary file 1 — Additional file 1. Guidelines for pre-departure presumptive malaria treatment for refugees resettling to the USA from sub-Saharan Africa. [file 12936_2018_2586_MOESM1_ESM.docx]

Additional File 1

Guidelines for pre-departure presumptive malaria treatment for refugees resettling to the USA from sub-Saharan Africa.

| Presumptive treatment pre-settlement in the USA for refugees from Sub-Saharan Africa | Pre-treatment Testing? | Medication |
| --- | --- | --- |
| **All adults and children weighing more than 5 kg (except pregnant and lactating women or known medication contraindications)*** | Treatment without testing | Artemether – lumefantrine |

***Pregnant women, lactating women, children weighing < 5 kg and those with other known contraindications are tested for malaria and treated if infection is found.**

See <https://www.cdc.gov/immigrantrefugeehealth/guidelines/domestic/malaria-guidelines-domestic.html>
