# Supplementary material for: Area of exposure and treatment challenges of malaria in Eritrean migrants: a GeoSentinel analysis
Source: Malar J. 2018 Nov 29;17:443. doi: 10.1186/s12936-018-2586-9 (PMC6267801; doi:10.1186/s12936-018-2586-9)
Supplement: Supplementary file 2 — Additional file 2. Availability of primaquine and G6PDH deficiency testing at GeoSentinel sites. [file 12936_2018_2586_MOESM2_ESM.docx]

Additional File 2: Availability of primaquine and G6PDH deficiency testing at GeoSentinel sites

| **Country** | **Is primaquine**  **licensed?** | **Primaquine availability** | **Availability of testing for G6PDH deficiency** | **Speed of results** |
| --- | --- | --- | --- | --- |
| Belgium | No | Available only through the Institute of Tropical Medicine, Antwerp | Available in hospitals and designated labs | Days |
| Norway | No | Has to be imported from abroad, some specialist units keep it in stock | Available through a designated lab | 7-14 days |
| Netherlands | No | Available via hospital pharmacies | Available | > 7 days |
| Denmark | No | Available via hospital pharmacies | Available | Days |
| Switzerland | No | In hospital pharmacies or with delay via the international pharmacy | Available through university hospitals and a designated lab | Hours or days depending on the urgency of the test |
| Germany | No | Has to be imported from abroad, some specialist units keep it in stock | Available through a designated lab in Bremen | 7-14 days |
| France | No | Available on special request and given free of charge | Available in university hospitals | Results in 4 hours |
| Sweden | No | Available from hospital pharmacies | Available | Result within one working day |
| Israel | No | Available from some hospital pharmacies. It is difficult to procure | Available | Days |
| United States | Yes | Available via pharmacies | Available | Tests are batched and results are available in days to weeks |
| Canada | Yes | Available via pharmacies | Available through reference labs | Quantitative tests may take weeks |
